# Spatial transcriptomics in neuroscience

**DOI:** 10.1038/s12276-023-01093-y

**Published:** 2023-10-02

**Authors:** Namyoung Jung, Tae-Kyung Kim

**Affiliations:** 1 Department of Life Sciences, Pohang University of Science and Technology (POSTECH), Pohang, Gyeongbuk 37673 Republic of Korea; 2 Institute for Convergence Research and Education in Advanced Technology, Yonsei University, Seoul, 03722 Republic of Korea

**Keywords:** Epigenetics and behaviour, Fluorescence in situ hybridization

## Abstract

The brain is one of the most complex living tissue types and is composed of an exceptional diversity of cell types displaying unique functional connectivity. Single-cell RNA sequencing (scRNA-seq) can be used to efficiently map the molecular identities of the various cell types in the brain by providing the transcriptomic profiles of individual cells isolated from the tissue. However, the lack of spatial context in scRNA-seq prevents a comprehensive understanding of how different configurations of cell types give rise to specific functions in individual brain regions and how each distinct cell is connected to form a functional unit. To understand how the various cell types contribute to specific brain functions, it is crucial to correlate the identities of individual cells obtained through scRNA-seq with their spatial information in intact tissue. Spatial transcriptomics (ST) can resolve the complex spatial organization of cell types in the brain and their connectivity. Various ST tools developed during the past decade based on imaging and sequencing technology have permitted the creation of functional atlases of the brain and have pulled the properties of neural circuits into ever-sharper focus. In this review, we present a summary of several ST tools and their applications in neuroscience and discuss the unprecedented insights these tools have made possible.

## Spatial transcriptomics: a new lens into brain function

Spatial transcriptomics technologies are revolutionizing our understanding of the brain’s complex cellular architecture and function. A recent review highlights the advances in imaging-based and sequencing-based methods that enable researchers to study gene expression at single-cell resolution within intact tissues. These tools have been applied to various areas of neuroscience, including mapping cell types in different brain regions, studying neural circuits, and investigating molecular and cellular responses to external stimuli. The integration of spatial transcriptomics with other cutting-edge techniques promises to provide unprecedented insights into the mechanisms underlying brain function and dysfunction, ultimately paving the way for new therapeutic strategies for neurological disorders.

## Introduction

The brain is one of the most complex organs, comprising highly diverse cell types intermingled in intricate anatomical structures, in which various subregions are responsible for specific functions. For example, the mammalian cerebral cortex is involved in motor and cognitive functions^1^, whereas the hypothalamus bridges the nervous system with the endocrine system through the pituitary gland^2^. Given that brain activity is exhibited in a region- or circuit-specific manner, understanding the cell-type composition and the spatial organization of individual cells is essential to decipher the role of these diverse cell types in brain function. In the past decade, a plethora of literature has reported diversely classified cell types in mouse and human brain tissues using single-cell RNA-seq (scRNA-seq) and single-nucleus RNA-seq (snRNA-seq)^3–7^. Although the census of molecularly distinct cell types in the brain greatly advances our understanding of the complexity of brain tissue, scRNA-seq requires intact cells to be dissociated from their native environment, resulting in a loss of spatial context^8^. The absence of spatial information for cell subclasses impedes the reconstruction of the cellular networks underlying various brain functions.

Spatial transcriptomics (ST) technologies permit the simultaneous mapping of cell types and their locations and have been applied in various biological contexts, such as embryo development, immune-cell responses to antigens, and different types of cancers^9–11^. From a technical point of view, the brain is the organ that benefits most from ST since the isolation of intact neuronal cells for scRNA-seq requires rigorous optimization of the dissociation protocol and delicate handling procedures to isolate cells from a region of interest^11,12^. On the other hand, many reference tools exist to guide neuroscientists in their anatomical and functional mapping endeavors, a benefit that is unique among commonly interrogated organs and tissues. The Allen Brain Atlas (ABA)^13^, for example, is a well-established repository of anatomical and molecular architecture, and molecular studies in the brain are also supported by the ample availability of scRNA-seq datasets, which have contributed to the initial establishment of ST tools in the mouse brain. Since the very first application of ST in the mouse brain, ST technologies have continued to evolve and adapt to more perplexing neurobiological questions, such as charting neuronal connectivity and discovering cell-type differences in neurodegenerative disease models compared with neurotypical brains^8^. Here, we discuss the current state of the art in ST and the novel findings yielded by the application of these tools in neurobiology.

## Technologies for spatial transcriptomics in current neuroscience

ST approaches in neuroscience have given the field a molecular cell atlas of various brain regions, mapped the network and interactions between neuronal cells and uncovered differences in cell states between normal and diseased brains. Each of the different available tools has unique capabilities and limitations with respect to field-of-view (FOV), scale, resolution, and detection efficiency, often with a trade-off among the parameters. As such, the knowledge obtained by these methods is technology-dependent. In general, technologies for ST can be classified into two groups: imaging-based and sequencing-based^8,10,12,14–16^. Imaging-based ST detects and measures RNA targets by in situ imaging via microscopy, which is further subdivided into in situ hybridization (ISH) and in situ sequencing (ISS)-based approaches, depending on how the target RNA molecules are identified and quantified. Sequencing-based ST adopts next-generation sequencing (NGS) of captured RNAs, predominantly polyA-tailed RNA species, from tissue sections. Imaging-based technologies offer high resolution and high efficiency at the single-cell or even subcellular level, while the resolution of sequencing-based methods mostly remains at the multicell level with lower detection efficiency (Table 1). Imaging-based modalities, however, are burdened by the inherent limitations of imaging in general, such as optical crowding, limiting the scale-up and expansion of the FOV. In addition, sequential rounds of hybridization require lengthy processing and analysis. Although sequencing-based approaches have lower detection efficiency and resolution than imaging-based approaches, they can be applied to a larger area; for example, very early examples of ST could profile an entire coronal section of the mouse brain, with the capacity to measure the whole transcriptome (Table 1)^17^. We will herein review the technical features of various ST tools used in neuroscience research.

**Table 1:** A summary of spatial transcriptomics technologies.

Method	Year	Throughput	Resolution	Pros	Cons
**Imaging-based**					
ISH					
seqFISH	2014	~250	Single cell	High detection efficiency High throughput	The small FOV Long imaging & processing time
seqFISH+	2019	~10,000			
MERFISH	2015	~10,000			
osmFISH	2018	33		Detecting lowly- expressed genes	Low throughput The small FOV
split-FISH	2020	317		High specificity No tissue clearing	Medium throughput The small FOV
EASI-FISH	2021	24		The applicability to 3D volume	Low throughput The small FOV
EEL FISH	2022	~440		The large FOV Short imaging time	Low detection efficiency
ISS					
STARmap	2018	~1020	Single cell	High signal-to-noise ratio The applicability to 3D volume	Low detection efficiency
BARseq	2019	NA		Mapping neuron projection	No gene expression
BARseq2	2021	65		Mapping neuron projection with gene expression	Low molecular throughput The small FOV
pciSeq	2020	99		The large FOV The computational assignment of cell identity	
FISSEQ	2014	Untargted		No reverse transcription	The small FOV
ExSeq	2021	Targted:42, Untargeted: ~1000		The nanoscale resolution	
ISH + ISH					
HybISS	2020	~120	Single cell	The large FOV	Low detection efficiency & throughput
**Sequencing-based**					
ST	2016	Untargeted	100 µm/ 55 µm	High accessibility The large FOV	Low resolution Low capture efficiency
Slide-seq	2019		10 µm	High resolution The large FOV	Multi-cell in some spots Low capture efficiency
Slide-seq V2	2021		10 µm		
HDST	2019		2 µm	High resolution The large FOV High sensitivity	
Stereo-seq	2022		0.22 µm		
Pixel-seq	2022		1 µm	Lower cost & less time for array production	
DBiT-seq	2020		10,25,50 µm	Multimodality	

### Imaging-based technologies

The ISH-based method quantifies RNAs via microscopic imaging of fluorescently labeled DNA probes that have been hybridized to their target RNAs. The ISS-based method detects individual target RNAs through rolling circle amplification (RCA) of padlock probes targeting RNA molecules and sequencing-by-synthesis or sequencing-by-ligation^8,11,12,14,18^.

#### ISH (in situ hybridization)

The ISH technique has a long history beginning in 1969^19,20^, progressing to the use of fluorescent labeling in 1977^21^. Single-molecule FISH (smFISH), developed in 1994, allows visualization of each transcript as a single spot under a microscope^22^ and is currently considered the gold standard method for quantifying RNAs within a cell. However, only a few target RNAs can be detected at a time due to spectral limitations.

**Fig. 1:**
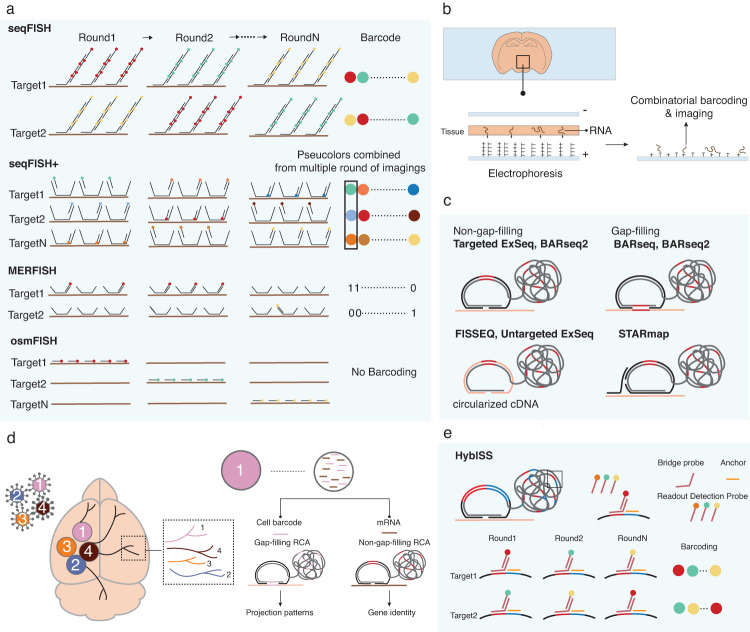
An overview of imaging-based spatial transcriptomics (ST) technologies used in neuroscience. **a** A schematic illustration of in situ hybridization (ISH)-based tools. Sequential FISH (seqFISH) identifies an RNA target by different combinations of fluorescent probes. An RNA is labeled with one color in each round of hybridization, enabling the colorimetric barcoding scheme. SeqFISH+ collapses multiple imaging rounds to generate ‘pseudocolor’ barcoding, in which an RNA is fluorescent in one of the sets of images and collapsed into one image corresponding to one round. Multiplexed error-robust FISH (MERFISH) exploits a combinatorial barcoding scheme in which a target RNA is assigned to a unique binary barcode, determined by on-off fluorescence in sequential rounds of hybridization. Cyclic-ouroboros FISH (osmFISH) does not use the combinatorial barcoding scheme but relies on multiple rounds of hybridization to label a target with a specific color in the image. **b** Enhanced electric FISH (EEL FISH). A tissue section is mounted on a capture slide coated with an electrically conductive layer of indium tin oxide (ITO). The ITO layer is modified with poly(D-lysine) and oligo(dT) to capture RNA electronically and chemically. The captured RNA is encoded and decoded by a combinatorial barcoding scheme. **c** A schematic illustration of the in situ sequencing (ISS)-based method. ISS-based techniques adopt various versions of padlock probes. Targeted expansion sequencing (ExSeq) and barcoded anatomy resolved by sequencing 2 (BARseq2) use non-gap-filling padlock probes, each of which contains a unique barcode. On the other hand, the gap-filling padlock probe used by BARseq and BARseq2 generates a barcode sequence complementary to a segment of the target sequence by gap-filling. Fluorescence in situ sequencing (FISSEQ) and untargeted ExSeq circularize cDNA, conferring padlock probe-like function on the cDNA. Spatially resolved transcript amplicon readout mapping (STARmap) uses SNAIL, in which a primer is hybridized to a target and provides a backbone to a padlock probe. The probes are amplified by rolling-circle amplification (RCA), generating a rolony containing a unique barcode for a target. In ISS-based technologies, fluorescent barcodes are generated by the incorporation of a fluorescent-conjugated nucleotide (sequencing-by-synthesis) or the ligation of fluorescent-conjugated oligonucleotides (sequencing-by-ligation). **d** BARseq2. BARseq2 can detect endogenous RNA expression and identify the projection pattern of the infected cell simultaneously. The neurons are barcoded by a viral infection, enabling the identification of projection patterns in different areas of the brain. The RNA barcode in a neuron is reverse transcribed and amplified by the gap-filling method. After amplification, the rolony containing the barcodes is sequenced to decipher neuron identity. The non-gap-filling approach is used for target gene detection. **e** Hybridization-based in situ sequencing (HybISS). HybISS integrates the advantages of ISH and ISS-based methods. HybISS uses the padlock probe and RCA to generate a rolony barcoded and decoded by a colorimetric scheme.

The current ISH-based methodologies exploit combinatorial barcoding, multiple rounds of hybridization, or both to improve the scalability of conventional smFISH. SeqFISH, the first scaled-up ISH, developed in 2014^23^, adopted both sequential hybridization and combinatorial barcoding. This technique uses the same probe sequences targeted to a transcript of interest through different rounds of hybridization but with a unique fluorophore in each round of imaging. In this way, each target gene is associated with a unique combination of fluorophores. The capacity of seqFISH is therefore theoretically determined by the number of fluorophores and imaging rounds (F^N^, F: number of fluorophores, N: number of hybridization rounds) (Fig. 1a and Table 1). This logic should permit whole-transcriptome profiling simply by increasing the number of hybridization rounds and fluorophores; however, seqFISH is limited in practice by optical crowding, which occurs when too many transcripts located in a densely packed cell are concurrently labeled and imaged^8,24^. To overcome such crowding issues, seqFISH+ leveraged 60 ‘pseudocolors’ with imaging in three fluorescent channels, which enables the detection of ~10,000 genes even at subcellular levels^25^. The technique employs primary probes containing four overhang sites for read-out probe hybridization, and complementary sequences to a target sequence are hybridized to target RNA. The fluorophore-labeled readout probes have a complementary sequence to one of the overhang sites in the primary probes. After the primary probes are hybridized to targets in cells, the sample is subjected to 20 rounds of readout-probe hybridization under one of the three channels. The images from the 20 rounds of hybridization are grouped to assign a pseudocolor to a gene in one round of sequential hybridization. Gene identity was decoded from a distinct combination of pseudocolors derived from four rounds, including one round for error correction (Fig. 1a)^25^.

Another imaging tool with high throughput is multiplexed error-robust FISH (MERFISH), which employs combinatorial barcoding and an error-correcting scheme, similar to seqFISH+, to allow the detection of ~10,000 genes in cell culture^26–29^. Each target is hybridized with ~192 encoding probes containing multiple arms for specific readout probes, rendering a distinct combination of readout sequences for each target (Fig. 1a and Table 1). Multiple readout probes correspond to one RNA transcript, and each readout sequence is detected in a specific hybridization round, returning a unique combination of binary barcodes for a target (the presence of the readout sequence in a hybridization round is 1, and its absence is 0). Each target gene possesses a unique combination of 0 and 1, with the bit number corresponding to the number of hybridization rounds^18,26–31^. While the sequential rounds of hybridization and imaging deployed in MERFISH dramatically enhance throughput, they can also by nature exponentially increase the misidentification rate, and most targets are not correctly assigned to their identity after 16 rounds of imaging. To circumvent the high risk of errors from multiple imaging rounds, MERFISH implemented a modified Hamming distance (defined as the minimum number of bits required to change one barcode into the other) codebook to increase the calling rate and reduce the error rate. For example, using a Hamming distance of four permitted the detection of 140 genes in 16 rounds of hybridization^27^. The MERFISH technique was recently commercialized as MERSCOPE by Vizgen, boasting a detection capacity of up to 500 genes, dramatically enhancing the accessibility of the MERFISH technique to the broader research community.

Although seqFISH and MERFISH greatly advanced the scalability of imaging-based ST, these tools still have disadvantages, such as the long time required for sequential imaging, the small FOV, the inability to provide 3D resolution, the high background signal, and the limitation of gene selection due to optical crowding or gene length (Table 1). Several other FISH-based tools have been developed to overcome these limitations. Ouroboros single-molecule FISH (osmFISH) only implements sequential rounds of hybridization, without combinatorial barcoding (Fig. 1a)^32^. osmFISH simply performs multiple rounds of smFISH. In this way, the images from osmFISH can be analyzed separately, enabling detection of low-expression targets and/or short genes that cannot be targeted by combinatorial barcoding methods. This technique still suffers from low detection throughput, limited to 33 transcripts in mouse and human brain tissues. Split-FISH offers improved specificity by reducing the background off-target signal by integrating the split-probe principle and multiplexed FISH^33^. In split-FISH, a pair of encoding probes will ideally bind to a target together to recruit a bridge probe, which will then be hybridized into dye-labeled readout probes. The high specificity of split-FISH enables bypassing a tissue-clearing step typically implemented in multiplexed FISH techniques to remove high background signal, hence greatly reducing sample preparation time. Expansion-assisted iterative FISH (EASI-FISH) can be applied to cleared thick tissue by physical expansion of the tissue section embedded in a swellable hydrogel^34^. The embedded tissue is digested, while the hydrogel simultaneously captures RNA through covalent attachment for tissue clearing. The captured RNA molecules are detected via multiround multiplexed FISH. Enhanced electric FISH (EEL FISH), enabling both a large FOV and high resolution, marries the advantages of both sequencing- and imaging-based ST tools. In this method, RNA molecules are transferred from a tissue section onto an electrically conductive slide coated with indium tin oxide (ITO) modified with oligo(dT) and positively charged poly(D-lysine), effectively rendering it an anode for capturing RNA. After RNA transfer to the slide and tissue removal by digestion, the captured RNA is decoded by 16 cycles of multiplexed FISH (Fig. 1b and Table 1)^35^. EEL FISH is currently under commercialization by Rebus Biosystem, awaiting a wider adoption of the technology in the research community.

#### ISS (in situ sequencing)

The concept of ISS, introduced in 2013, leverages three methods: padlock probing, rolling-circle amplification (RCA), and sequencing-by-ligation^36^. The padlock probe contains sequences at its 5’ and 3’ ends that are hybridized to target cDNA and can be used in two targeted ways: gap-filling and non-gap-filling (Fig. 1c). In the gap-filling method, the padlock probe binds to the cDNA with a gap between the 5’ and 3’ ends of the probe, in which the gap corresponding to a segment of target cDNA is filled and amplified by DNA polymerase and ligase (Fig. 1c). The padlock probe used for the non-gap-filling scheme carries a unique barcode for each target sequence (Fig. 1c). These padlock probes are replicated by RCA, which generates concatenated copies of the probes. The resulting rolling circle product (RCP), or “rolony,” is then subjected to sequencing by sequencing-by-ligation or sequencing-by-synthesis chemistry^8,36^.

STARmap (Spatially resolved transcript amplicon readout mapping) modifies ISS by applying hydrogel-tissue chemistry, SNAIL probes, and sequencing with error-reduction by dynamic annealing and ligation (SEDAL), enhancing its efficiency in intact tissue relative to conventional ISS (Fig. 1c and Table 1)^37^. To increase the detection efficiency of ISS, SNAIL bypasses reverse transcription, the major rate-limiting step in ISS, by using a pair of primers and padlock probes that directly bind to mRNAs. The primer offers a template for padlock ligation, such that the padlock probes are amplified only when the primer cobinds to the target mRNA, producing an amplicon (Fig. 1c). After the rolonies are formed, the tissue is cleared with hydrogel-tissue chemistry^38^ and subjected to SEDAL. SEDAL reads the five-base barcode on the padlock probe with two-base encoding, which facilitates error correction.

The high spatial resolution of ISS in tissues lends advantages to the technique for specific biological questions. For example, in neuroscience, deciphering neuronal connectivity across different brain regions is critical to understanding neural circuits. To interrogate connectivity by ST, barcoded anatomy resolved by sequencing (BARseq) leverages the principles of ISS with neuron barcoding and read-out by gap-filled padlock probes (Fig. 1c, Table 1)^39^. The RNA barcodes are delivered by the sindbis virus into the target region in the brain and are then decoded by ISS. The recently introduced upgraded version of BARseq, BARseq2, enables simultaneous mapping of neuronal projections and gene expression in the barcoded neuron by applying a combination of gap-filled padlock probe technology for projection mapping and non-gap-filled padlock probes targeting endogenous mRNAs (Fig. 1c, d)^40^.

While ISS can provide high signal-to-noise data through RCA, difficulties in delineating cell boundaries based on 2D imaging limit its ability to assign profiled genes to specific cells. Probabilistic cell typing by in situ sequencing (pciSeq) overcomes this limitation through a computational approach based on probabilistic modeling^41^. Although this method prerequires scRNA-seq data for the tissue of interest, it offers an efficient method of cell-type profiling in a relatively large FOV (Table 1).

ISS can also be carried out in an untargeted way by circularizing the cDNA itself in a similar fashion to how a padlock probe is generated in a targeted way for RCA to create the rolony. In fluorescence in situ sequencing (FISSEQ)^42,43^, cDNA fragments are circularized and amplified by RCA, followed by sequencing (Fig. 1c). Moreover, expansion sequencing (ExSeq) also has an untargeted strategy that combines FISSEQ and expansion microscopy to achieve higher efficiency and resolution in tissue (Fig. 1c and Table 1)^44^. The accessibility of ISS-based methods is expanding, as are ISH-based tools. The original ISS technique became commercialized as CartaNA, which was acquired by 10x Genomics in 2020 and provided the basic principles for Xenium, an in situ ST platform newly released by 10X Genomics^11^.

Each ISH- and ISS-based approach has its own advantages: combinatorial barcoding, allowing upscaling for ISH, and high specificity in a large area for ISS. Hybridization-based in situ sequencing (HybISS) combines the strengths of both tools by adopting the padlock probe and RCA methodologies from ISS and the highly multiplexed barcoding of ISH^45^. In HybISS, the target cDNA is bound by padlock probes amplified by RCA, producing a rolony, and then sequentially imaged and decoded as in MERFISH or seqFISH. To enable combinatorial barcoding, the padlock probes in HybISS include a gene-specific ID sequence and anchor sequences that are universal among a subset of the probes. After a rolony is formed through probe amplification, a bridge probe hybridizes to a gene-specific ID sequence, and a fluorophore-conjugated readout detection probe binds to the bridge probe. This process is repeated in sequential cycles to generate a unique colorimetric barcode for a given gene (Fig. 1e).

### Sequencing-based technologies

**Fig. 2:**
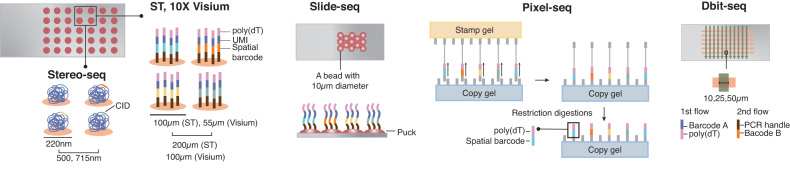
A schematic illustration of sequencing-based technologies. In the original ST, capture probes containing poly(dT), unique molecular identifier (UMI), and a spatial barcode are printed in an array of spots with 100 μm in diameter. The ST is commercialized as Visium by 10X Genomics with a resolution of 55 μm in diameter. In spatiotemporal enhanced resolution omics sequencing (Stereo-seq), a DNA nanoball including a spatial barcode is printed on the slide as a 220 nm diameter spot, providing the highest resolution among sequencing-based techniques. Slide-seq uses a bead with a 10 μm diameter instead of a spot, forming a layer called a “puck” on a slide. Polony-indexed library sequencing (Pixel-seq) generates capture-probe printed gels with the same arrangements of the probes across different gels, hence greatly reducing the time and cost of producing and decoding spatially barcoded arrays. Deterministic barcoding in tissue for spatial omics sequencing (DBiT-seq) enables the simultaneous mapping of proteins and RNA. The capture probes are delivered to tissue by a microfluidic device in two flows. The first flow contains probes with poly(dT) and spatial barcode A, and the second flow delivers another probe containing spatial barcode B perpendicular to the first flow. The two barcodes A and B are joined at the intersection of the two flows, which forms a pixel with a unique combination of barcodes A and B. The resolution of DBiT-seq is 10, 25, and 50 μm, determined by the channel width of a microfluidic device.

In parallel with the development of imaging-based tools for ST, sequencing-based methodologies have also evolved over the past decade. The term “spatial transcriptomics” was coined by the first NGS-based study to spatially map RNA molecules in an intact tissue, which also introduced the concept of spatial barcoding in two dimensions^17,46^. In its original iteration, ST comprised an array of spots coated with reverse-transcription oligo(dT) primers with positional molecular barcodes printed on a glass slide, enabling unbiased capture of mRNA at the whole-transcriptome level and the capturing of spatial information (Fig. 2). A fixed tissue section was mounted on the slide and permeabilized to allow diffusion of RNA molecules from the tissue to the slide. These RNAs were then captured by spatially barcoded primers and reverse transcribed to generate complementary DNA (cDNA), which was then subjected to sequencing. The resolution of the initial ST was 100 μm of spot diameter, with any given spot encompassing transcriptome information from multiple cells^47^. This method was commercialized by 10X Genomics and branded Visium, with a 55 μm spot diameter and a higher resolution than the original method. Despite its relatively low resolution compared with imaging-based tools, ST has several benefits: covering large tissue sections, including whole mouse brain^48^; unbiased detection of mRNA at the whole transcriptome level; and wide availability through commercialization (Table 1).

Slide-seq improved the resolution of sequencing-based methodologies by using DNA-barcoded beads with a 10 μm diameter deposited onto a rubber-coated glass coverslip, forming a tightly packed monolayer called a “puck” (Fig. 2)^49^. The spatial barcodes on the beads are generated through split-pool barcoding, a protocol used for single-cell barcoding^50^, which is determined prior to mounting tissue sections onto the puck. A tissue cryosection is melted onto the puck and transferred to an Eppendorf tube, in which sequencing libraries are generated. The spatial index on the beads and mRNAs captured from a tissue section are sequenced using SOLiD chemistry^49^. Although Slide-seq has greatly improved the resolution of ST, it exhibits relatively low RNA capture efficiency, ~5% of what scRNA-seq typically achieves (Table 1). To improve its low sensitivity, Slide-seqV2 was developed by modifying bead indexing and library generation, achieving ~10-fold higher capture efficiency than the original technique^51^.

High-definition spatial transcriptomics (HDST), a method similar to Slide-seq in its use of a barcoded bead array, allows for higher resolution by using beads that are smaller than single cells. The barcoded beads are enriched with poly(dT) nucleotides and deposited into 2μm wells to obtain high resolution^52^. Both the Slide-seq and HDST methods offer high resolution with spatial mapping of the transcriptome mostly at the cellular level, although many spots contain multiple cells.

The extensive effort to achieve single-cell or even subcellular resolution with ST culminated in the development of spatially enhanced resolution omics sequencing (Stereo-seq), which offers submicron (0.22 μm) resolution (Table 1)^53^. The high resolution of Stereo-seq is afforded by DNA nanoball (DNB)-mediated in situ sequencing (Fig. 2). The DNB, produced by RCA and printed on an array, has a diameter of 220 nm (center-to-center distance of 500–715 nm) and contains random barcodes carrying the spatial index, called the coordinate identity (CID). Next, oligonucleotides containing a unique molecular identifier (UMI) and poly(dT) are introduced to each spot by hybridization with random primers containing the CID. Tissue is mounted on the DNB-patterned array, and the polyA-tailed RNAs are captured, reverse-transcribed, and amplified. The libraries of these cDNAs are prepared and sequenced together with the spatial index. In addition to its nanometer-scale resolution, Stereo-seq offers a large FOV of up to 200 mm^2^, ~5-fold larger than the area covered by Visium.

Most sequencing-based tools use arrays of primers containing spatial barcodes that require decoding by additional sequencing for each array, increasing the cost of the assay and hindering the scale-up of array production. Recently, polony-indexed library sequencing (Pixel-seq) has been developed to tackle this issue by adapting a polony-gel stamping method with 1 μm resolution^54^. A stamp gel is first generated by forming polonies, DNA clusters containing poly(dT), and spatial barcodes through bridge amplification and linearization on the surface of crosslinked polyacrylamide. The polonies on the stamp gel are replicated on a copy gel by a polymerase-catalyzed chain reaction, which is repeated to produce many copy gels with the same spatial barcode information (Fig. 2 and Table 1).

The sequencing-based tools were expanded to map transcripts and proteins concurrently. Deterministic barcoding in tissue for spatial omics sequencing (DBiT-seq) adopts microfluidics to generate pixels of 10, 25, or 50 μm resolution even in formalin-fixed, paraffin-embedded (FFPE) tissue^55^. In DBiT-seq, a microfluidic device with 50 parallel microchannels is placed on a tissue section and delivers DNA barcodes bearing spatial index and oligo(dT) sequences (Fig. 2 and Table 1). The first set of barcodes initiates in situ reverse transcription during the first flow, and then the first chip is removed. Another microfluidic chip is placed on the tissue and introduces a second set of DNA barcodes containing distinct spatial barcodes, UMI, and biotin perpendicular to the first flow of barcodes, generating a mosaic of tissue pixels with a specific combination of the two barcodes. The resolution of DBiT-seq is determined by the size of the channel width, which can currently be as small as 10 μm. Tissue morphology is aligned to the spatial map by imaging the slide during the flows or afterward. Mapping the proteins in DBiT-seq is carried out similarly to CITE-seq^56^, a scRNA-seq method combined with protein profiling, before flow barcoding. After creating mosaic pixels with cDNA and spatial barcoding, libraries are generated and sequenced to reconstruct a spatial omics map of RNA and proteins based on the barcode combination and tissue imaging.

The sequencing-based tools summarized above offer several advantages over imaging-based technologies, such as a larger FOV, i.e., a whole mouse embryo with Stereo-seq; a high potential for commercialization, which broadens accessibility to the techniques as seen in commercialization efforts such as Visium; shorter processing time without multiple rounds of imaging; and unbiased selection of the whole transcriptome as long as the target molecule has polyA tails. However, these techniques also have some drawbacks. Although they claim to achieve single-cell and even subcellular resolution, the sequencing data in fact come from a single-cell or subcellular region-sized spot and not necessarily from a single cell itself. Furthermore, the capture chemistry used in most sequencing-based tools exhibits low RNA detection efficiency, therefore resulting in much lower sensitivity relative to imaging-based methodologies (Table 1)^8,11,12^. Thus, it is critical for researchers to select the most appropriate tool to address their questions adequately.

## The application of spatial transcriptomics in neuroscience

**Fig. 3:**
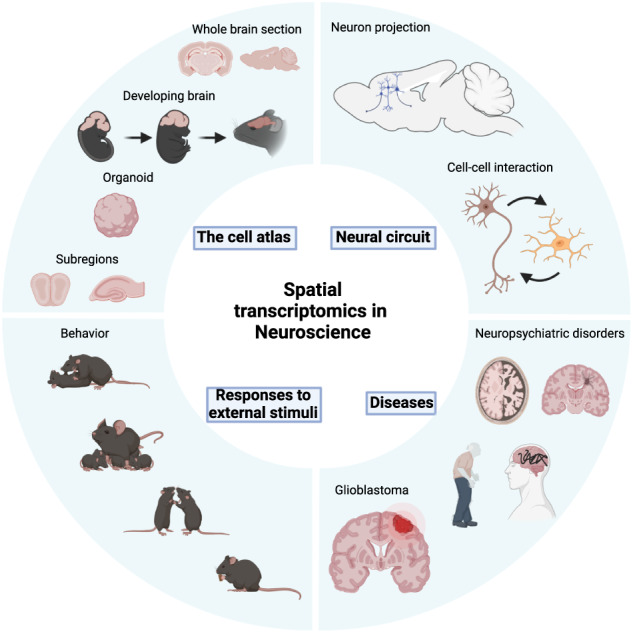
The application of ST in neuroscience. In neuroscience, ST has been employed to generate a cell atlas of various brain regions and has also been used to map neural circuits, to investigate the molecular and cellular responses to external stimuli and to characterize the differences between normal and diseased brains. (Created with BioRender.com).

In the past decade, collective efforts by several large-scale initiatives to generate cell atlases, such as the BRAIN Initiative Cell Census Network (BICCN) and the Human Cell Atlas, have greatly expanded our understanding of cell-type compositions in various tissues^57,58^. Recent advances in ST technology have started adding a new dimension of knowledge to cell-type mapping efforts, especially in the brain^59^. In this section, we will discuss the findings from ST approaches in the following areas: the spatially-resolved brain cell types, neural circuits, and brain activity in diverse contexts, including behavior and diseases (Fig. 3).

### The spatial cell atlas of the brain

As mentioned above, several ST methods were first adopted and validated in the brain, simultaneously providing cell-specific profiling and spatial information. SeqFISH and MERFISH (imaging-based methods) and 10X Visium (sequencing-based method) are the most frequently applied techniques to examine the cell types in subregions of the brain, primarily in mouse models.

One of the initial applications of imaging-based approaches explored cell types in subregions of the mouse hippocampus, temporal cortex, and parietal cortex by seqFISH, targeting ~250 genes and identifying 13 transcriptionally distinct cell clusters^60–62^. The study found that a unique combination of cell populations constituted the hippocampal subregions, particularly CA1 and CA3. Increased cell-type heterogeneity was observed in the ventral area compared to the dorsal area. However, seqFISH was not able to distinguish various subpopulations of interneurons due to the limited number of genes that can be interrogated (only 100-200 genes). With the upgraded version of seqFISH, called seqFISH+, the target‒gene number was scaled up from hundreds to 10,000 genes and was implemented for cell-type profiling of the mouse cortex, subventricular zone (SVZ), and olfactory bulb, identifying diverse, region-specific cell types concordant with scRNA-seq results^25^. Furthermore, the subcellular-level spatial information provided by seqFISH+ enabled the examination of tissue-specific ligand-receptor interactions, which was not feasible in dissociated-cell experiments. For example, spatial profiling revealed that endothelial cells adjacent to microglia expressed type I and III TGF β receptors, and microglia expressed Tgfb1 in the olfactory bulb, while endothelial cells adjacent to microglia in the cortex harbored Lrp1 and Pdgfb, revealing the significance of the local spatial context of neighboring cells for ligand‒receptor expression patterns in a specific cell type. As seqFISH also allows for the study of chromatin structures by using probes targeting genomic regions of interest instead of RNA sequences^63,64^, seqFISH and seqFISH+ were further expanded to multiomics approaches in the mouse cerebral cortex, in which DNA seqFISH+ and RNA seqFISH were performed for 3D genome folding analysis and spatial gene expression analysis, respectively^65^. The integrated spatial omics approach deployed here revealed that the distinct cell subclasses harboured specific nuclear architecture that correlated with different gene expression patterns in the different cell types.

Among the imaging-based techniques, MERFISH is the most frequently used in studies that spatially profile cell types in a variety of subregions of the mouse brain^29,30,59,66–69^. Moffitt et al. investigated the distinct neuronal cell types in hypothalamic nuclei using MERFISH targeting 155 genes along with scRNA-seq^30^. This combined approach led to the discovery of ~70 neuronal cell types: ~30 excitatory and ~40 inhibitory neurons in the hypothalamic preoptic region. In addition to observing the unique spatial segregation of these clusters in the preoptic region, the high sensitivity of MERFISH has permitted the analysis of weakly expressed genes, such as the receptors of neuromodulators and hormones, which play important roles in hypothalamic function. Cell types underlying social interactions, which are particularly amenable to interrogation with techniques such as MERFISH due to the subtlety of their expression profiles, were thus spatially mapped in the hypothalamus. Using MERFISH, BICCN and others allowed for the exhaustive characterization of spatial cell-type profiles in the mouse primary motor cortex (MOp)^29,59,66^. By assessing 258 genes, including canonical marker genes for major neuronal and non-neuronal cell types in the cerebral cortex, MERFISH identified a total of 95 neuronal and non-neuronal cell clusters: 39 excitatory, 42 inhibitory, and 14 non-neuronal clusters in the MOp^29,59^. The study also uncovered the laminar pattern of GABAergic neurons and the gradient distribution of intratelencephalic (IT) neurons across different layers of the cortex. As isoforms of genes are often shown to be specific to cell types in the mouse brain^4,66,70^, the integration of gene isoform expression from SMART-seq and the spatial information of the marker genes from MERFISH have been invaluable in the generation of the spatial gene isoform atlas in the mouse MOp. This integrated approach illustrates a wider application of ST to identify novel, cell type-specific markers in combination with other technologies^66^. In the mouse striatum, MERFISH identified 8 cell clusters, including D1 and D2 medium spiny neurons (MSNs), the major cell types in the striatum, by targeting 253 genes^67^. The D1 and D2 MSN cells were further segregated into 15 D1 and nine D2 subclasses, showing spatial heterogeneity along the anterior-to-posterior (AP) axis. The improvement of molecular throughput in MERFISH has allowed the targeting of 4000 genes and identification of 125 cell clusters in the human middle temporal gyrus (MTG)^68^. Moreover, comparative analysis of human and mouse cortices has highlighted the substantial cell composition differences between human and mouse brains; for example, a lower proportion of excitatory neurons and a higher proportion of other cell types, including oligodendrocytes, oligodendrocyte progenitor cells, and mural cells, were observed in the human cortex compared to the mouse cortex. Although the cells in both organisms’ brains showed a conserved laminar distribution, MERFISH revealed a differential spatial pattern for some cell types. For instance, L6b neurons were dispersed in layers 5 and 6 and white matter in humans, whereas this neuronal subtype was restricted exclusively to the bottom of L6 in mice. The high resolution of MERFISH also enabled cell‒cell interaction analysis by permitting inference of interactions based on the proximity of different cell types to each other. Interestingly, the analysis showed a higher frequency of contacts between neuronal and non-neuronal cells, such as neuron-oligodendrocytes and microglia-excitatory neurons, in the human cortex relative to mice, indicating the evolutionary adaptation of human neuronal cells to meet higher energy demand and maintain complex tissue homeostasis.

MERFISH has also been used to examine how the spatial distribution of a particular cell type affects other cells in the mouse somatosensory cortex, focusing on the spatial relationship between projection neurons (PN) and microglia^69^. Interestingly, the various subtypes of microglia could be broadly divided into two types depending on their spatial patterns relative to PN subtypes: PN subtype-responsive microglia exhibited specific laminar localization, as in PN subtypes, whereas PN subtype-insensitive microglia were dispersed along the laminar layers. The presence of PN subtype-responsive microglia suggests a novel role of neuronal diversity in shaping the microglial states that calibrate neuroimmune interactions in the niche. In addition to cell-type mapping, MERFISH can be performed to validate a predictive model for the locations of cell clusters, such as glomeruli^71^.

Other ISH-based methods with different strengths have been established and applied in the brain. By targeting a smaller set of genes relative to other methods that use combinatorial barcoding but include a critical gene set for cell types of interest, osmFISH identified 31 clusters in the mouse somatosensory cortex with 31 marker genes^32^. Later, osmFISH was applied to validate neuronal markers for distinct cell types in six areas of the developing human cortex, confirming the laminar expression pattern of the 31 genes. The feasibility of split-FISH probing 317 randomly selected genes was assessed and demonstrated in mouse brain tissue^33^. The utility of EASI-FISH in thick tissue was demonstrated in a study that focused on the lateral hypothalamic area (LHA) in 300 μm-thick coronal mouse brain slices^34^. EEL FISH was applied to the whole mouse brain of eight sagittal sections, generating a mouse brain atlas by measuring ~440 genes. EEL FISH was also used to assess the expression of 445 genes in the human primary visual cortex, successfully illustrating layer-specific expression and cell-type positioning in this structure^35^.

Among the ISS-based tools, FISSEQ was the first method established in mouse brain tissue^43^. Following the successful implementation of FISSEQ, STARmap was developed and used to build a cellular atlas of mouse and chicken brains^37,72^. In the first published example of STARmap, Wang et al. analyzed 160 and 1020 genes in the mouse prefrontal cortex (mPFC) and visual cortex, respectively, and successfully revealed the differences in cell-type composition between the two regions. For example, eL4 excitatory neurons accounted for one-third of the excitatory neurons in the visual cortex but were not present in the mPFC^37^. A comparative study between mouse and chicken cerebellar nuclei was carried out by STARmap combined with snRNA-seq, identifying a spatially distinct population composed of two excitatory and three inhibitory classes that were conserved between mouse and chicken through duplication during evolution^72^.

Another ISS-based tool, pciSeq, which refined the ISS by leveraging a probabilistic model to computationally assign cell types, mapped different types of inhibitory neurons in the hippocampal region and revealed laminar expression patterns concordant with previous works^41^. ExSeq can operate in either an untargeted or targeted manner, as described previously^44^. Untargeted ExSeq was performed on 15- and 50 μm-thick mouse hippocampal slices and used to determine the dendritic localization of intron-retained transcripts in hippocampal neurons. ExSeq targeting 42 genes in the mouse visual cortex revealed the laminar pattern of distinct cell clusters. In addition, by analyzing 34 RNA molecules previously found to be localized in dendrites, ExSeq demonstrated a neuronal compartment-dependent transcription pattern. HybISS, a method incorporating the advantages of both ISH and ISS, was used to map ~120 genes in mouse and human brain^45^. In mice, a whole coronal section of an adult mouse brain was efficiently mapped, identifying distinct cell clusters with layer-specific distribution in cortical tissue. For the human brain, HybISS was performed on the middle temporal gyrus, showing the laminar spatial pattern of marker genes, as found in the mouse brain. Later, HybISS was applied in an E10.5 mouse embryo to provide a spatial map of the developing mouse brain by refining cell clusters derived from scRNA-seq^73^.

A sequencing-based ST method developed by the Lundeberg group was first demonstrated in the mouse olfactory bulb^17^, where the anatomy is well defined and ample gene expression data are available. Later, other sequencing-based technologies, such as Slide-seqV2, HDST, Stereo-seq, and Pixel-seq, were implemented in the mouse olfactory bulb using the Lundeberg ST data as a reference^51–54^. Moreover, ST was adopted to generate a molecular atlas of the whole mouse brain, identifying 181 molecular and structural clusters of cell types by examining the whole transcriptome. This study also defined a set of 266 genes, called a ‘brain palette’, from which 181 clusters were reconstituted^48^. Visium, a commercialized ST platform, showcased the power of its spatial profiling technology in the human brain, particularly the dorsolateral prefrontal cortex (DLPFC)^24,74^. This study discovered novel layer-specific markers, including HPCAL1 for L2 and KRT17 for L6, as well as layer-specific enrichment of genes related to neurodegenerative diseases, indicating the clinical importance of spatial gene expression patterns. Visium has been the most commonly used technique in neuroscience thus far, reporting ~40 publications utilizing fresh-frozen brain tissues from diverse organisms.

Slide-seq, which has higher spatial resolution than Visium, was exploited in the mouse cerebellum and hippocampus to successfully extract spatial information on scRNA-seq-defined cell types for the two regions^49^. Slide-seqV2 has been widely used to examine the mouse brain in various biological contexts, including the developing mouse brain, hippocampus, olfactory bulb, cerebral cortex, and brain organoid^51,71,75,76^. The first application of Slide-seqV2 demonstrated its capacity to reconstruct developmental trajectories in the E15 mouse cortex^51^. Similarly, Slide-seqV2 has reconstructed a comprehensive spatial cell atlas and differentiation trajectory in E12.5, E13.5, E15.5, and P1 mouse neocortices undergoing corticogenesis using a computational method called Tangram^77^, which integrates the spatial data from Slide-seqV2 and scRNA-seq^75^. Slide-seqV2 has also pinpointed previously unknown glomeruli locations in the mouse olfactory bulb by identifying proximal spots expressing the same olfactory receptors^71^. The development of human brain organoids was comprehensively tracked with Slide-SeqV2 and other omics tools, including scRNA-seq, scATAC-seq, and SHARE-seq, revealing a high correlation between organoid development and endogenous events^76^.

HDST accurately captured layer-specific gene expression patterns in the mouse olfactory bulb at near single-cell resolution^52^. Recently, at even higher resolution, the mouse olfactory bulb was profiled by Stereo-seq^53^ and Pixel-seq^54^. The robustness of the Stereo-seq method was validated by profiling mouse coronal hemibrain, successfully recapitulating the cell types discovered in the scRNA-seq analysis and revealing the spatial location of the clusters. Furthermore, the large FOV of Stereo-seq allowed the generation of the mouse organogenesis spatiotemporal transcriptomic atlas (MOSTA) by interrogating whole mouse embryos at different time points^53^. Pixel-seq was used to create the first spatial cell atlas of the mouse parabrachial nucleus (PBN) and pain-regulated changes in the spatial transcriptome in PBN^54^.

DBiT-seq, a high-resolution, multimodality sequencing-based method, was used to examine the whole transcriptome of the E10 mouse embryonic brain, identifying 22 proteins enriched in the forebrain and microvasculature^55^. Whole transcriptome analysis identified 11 distinct clusters enriched in biological processes critical to embryonic brain development, such as telencephalon development and regionalization.

### The spatially resolved brain in various biological contexts

In the field of neuroscience, ST is increasingly being used to delve into more complex biological questions. How are neural circuits constructed from among distinct cells in the same region or across different regions of the brain? How do these distinct cell types respond to diverse external stimuli? What are the differences in the spatial composition of cell types between normal and diseased brains? This section will illustrate several examples of ST-based studies in various contexts of neurobiology (Fig. 3).

The functional mechanisms of the brain depend on the cellular connectivity that constitutes the neural circuit. Mapping neural circuitry at single-cell resolution with high throughput requires the development of novel methods such as BARseq. BARseq has integrated multiplexed analysis of projections by sequencing (MAPseq)^78^, a method for mapping projections of neurons independent of spatial information, and ISS^39^. BARseq has examined neuronal projections from ~3600 neurons in the mouse auditory cortex to 11 target areas, including most major regions known to be projected by the auditory cortex. The BARseq results recapitulated the preestablished laminar distribution of the three subtypes of cortical projection neurons, including IT, pyramidal tract-like (PT-like), and corticothalamic (CT), and discovered 25 clusters of diverse projection patterns. Interestingly, BARseq identified IT subtype-specific projection patterns through an approach that also incorporated scRNA-seq. The improved version of BARseq, BARseq2, was capable of detecting gene expression and projection patterns simultaneously. The method is thus capable of correlating projection patterns with the expression of cell-type markers, including the cadherin gene, in the mouse motor cortex and auditory cortex^40^. It was revealed that projections of IT neurons to the two cortices were mediated by a shared set of cadherins, demonstrating the potential of BARseq2 to unveil the molecular grammar underlying neural circuits.

A fundamental puzzle in neurobiology is how distinct cell types within a region render a specific function to the subregion in response to a specific external stimulus. For example, the hypothalamus governs diverse social behaviors, yet the mechanism whereby different cell types in the region molecularly react to social cues to effect behavioral changes remains unknown. Using MERFISH, Moffit et al. examined how transcriptionally distinct cell types in the mouse hypothalamus impart the functional differences that underlie diverse social behaviors, such as parenting, mating, and aggression. The MERFISH targeting cFos, a representative immediately early gene (IEG) for a neuronal activation marker, showed that a specific combination of neuronal clusters varying by sex, age, and virginity was involved in a specific behavior. For example, two inhibitory neuron clusters, I-15 and I-16, were activated in female mice, while five inhibitory neuron types (I-2, I-11, I-14, I-15, and I-33) and two excitatory cell types (E-8 and E-15) were active in male mice during mating^30^.

Among neurodegenerative diseases, amyotrophic lateral sclerosis (ALS), Alzheimer’s disease (AD), and multiple sclerosis (MS) have been thoroughly studied using ST by several groups^79–81^. ST was initially implemented to construct a comprehensive spatiotemporal whole-transcriptome map for ALS. This study identified signaling pathways dysregulated during the presymptomatic phase, differential spatiotemporal expression patterns of specific cell types, including astrocytes and microglia, in a mouse ALS model, and a common dysregulation of sphingolipid signaling in both mouse models and human patients^79^. ST also identified two gene modules enriched in the local amyloid plaque niche in a mouse model of AD: plaque-induced genes (PIG), which changed in multiple cell types, and an oligodendrocyte gene (OLIGs), which was associated with myelination and was enriched in oligodendrocytes. This result was recapitulated in human AD patients using ISS, supporting the clinical relevance of these modules^80^. Furthermore, gray matter tissues of MS patients were also rigorously interrogated by ST. Tracking of the mechanisms underlying neuronal degeneration in MS identified altered synapse biology gene modules across different stages of neurodegeneration and indicated failure of anti-inflammatory intercellular communication in the early phases of neurodegeneration^81^. ST has also provided mechanistic insight into neurodevelopmental and neuropsychiatric illnesses. Visium has confirmed previously reported snRNA-seq data and histological studies that reveal transcriptional and cell-type compositional alterations in upper cortical layers in the DLPFC of schizophrenia patients. Synaptic transmission and organizational pathways were found to be dysregulated, and a subset of GABAergic and principal neurons showed a significant compositional change in the upper layers of the DLPFC^82^.

Glioblastoma (GBM), a type of brain cancer, was also spatially resolved by MERFISH and Visium^83,84^. Hara et al. demonstrated the critical role of the interaction between macrophages and glioblastoma cells in the transition of cancer cells into mesenchymal-like (MES-like) states by integrating ST, scRNA-seq, and other functional studies^84^. In this study, MERFISH targeting 135 marker genes for MES-like glioblastoma and macrophage cells was carried out in human GBM samples. The results were consistent with findings from a mouse GBM model that showed enrichment of macrophages in the vicinity of MES-like GBM cells. Visium analysis of GBM samples from 20 patients, along with spatially resolved metabolomics and proteomics, demonstrated that five spatially distinct transcriptional programs could be segregated based on the characteristics of upregulated genes: radial glia, reactive immunity, spatial OPC, neuronal development, and reactive hypoxia^83^. Interestingly, reactive hypoxia occupied a special niche enriched for chromosomal aberrations, suggesting a strong correlation between altered metabolism and genomic instability. Additionally, the reactive-immune cluster showed enhanced cellular interactions between the immune and cancer cells in cellular interdependence analysis. These results advanced our understanding of the relationship between the microenvironment and dysregulation in the spatially resolved transcriptional heterogeneity of GBM.

Recently, the application of ST has expanded to encompass many different biological contexts. For example, MERFISH was applied to define the spatial signatures of aging and fever in the mouse brain^85,86^, and Stereo-seq resolved spatial single-cell transcriptomics during the development and regeneration of the axolotl brain^87^. Recent research trends suggest that ST has the power to underwrite the creation of a complete spatiotemporal cell atlas for a wide range of neurological processes in a variety of organisms.

## Concluding remarks

In this review, we have summarized ST techniques and their specific applications in neuroscience, as well as the groundbreaking insights gained from these approaches in diverse neurobiological contexts. ST has been adopted across a wide variety of neuroscience subdisciplines, from molecular single-cell profiling in a spatial context to functional studies of neurological disease, including brain cancer. The rapid advances in ST methods continue to unveil mechanisms underlying complex neurobiological processes beyond spatial cell-type profiling in the brain. For example, BARseq has enabled the mapping of neuron projections, revealing neuronal network architecture in rich detail^39^, and MERFISH captured cell‒cell interactions by examining cell‒cell proximity and ligand‒receptor interactions^88^. Exemplified by DBiT-seq^55^, capable of simultaneous spatial profiling of the whole transcriptome and target protein expression, enhanced ST modalities have begun to dissect the multilayered molecular mechanisms of neurological questions. Moreover, the recent divergence of the ST tools listed above invites the interrogation of other modalities, including epigenomics by MERFISH^89^, 3D chromatin looping by ISH-based tools^63^, and genomics by Slide-seq^90^, implicating future utility for these technologies in the exploration of various aspects of neurobiology. Similar to BARseq, the integration of spatial transcriptomic approaches with current-era neuroscience techniques, such as optogenetics and calcium imaging, opens new avenues by which to approach the most challenging neurobiology questions. The technical revolution in ST will shed unprecedented light on the mechanisms whereby the brain orchestrates neuronal function, circuitry, and behaviour.
